# Supplementary material for: COZOID: contact zone identifier for visual analysis of protein-protein interactions
Source: BMC Bioinformatics. 2018 Apr 6;19:125. doi: 10.1186/s12859-018-2113-6 (PMC5889581; doi:10.1186/s12859-018-2113-6)
Supplement: Supplementary file 2 — Software build. Executable binary file of the software tool. (ZIP 118,784 kb) [file 12859_2018_2113_MOESM2_ESM.zip › cozoid/platform/docs/testng-6.5.1-javadoc.zip!/org/testng/SuiteRunnerWorker.html]

SuiteRunnerWorker (TestNG)


---


|  |  |  |
| --- | --- | --- |
| **Overview** \| **Package** \| **Class** \| **Tree** \| **Deprecated** \| **Index** \| **Help** | |  |
| **PREV CLASS**   **NEXT CLASS** | **FRAMES**    **NO FRAMES**     **All Classes** |  |
| SUMMARY: NESTED \| FIELD \| CONSTR \| METHOD | DETAIL: FIELD \| CONSTR \| METHOD |  |


---


## org.testng Class SuiteRunnerWorker

```
java.lang.Object
  org.testng.SuiteRunnerWorker
```

**All Implemented Interfaces:**: java.lang.Comparable<org.testng.internal.thread.graph.IWorker<ISuite>>, java.lang.Runnable, org.testng.internal.thread.graph.IWorker<ISuite>

---

``` public class SuiteRunnerWorker extends java.lang.Object implements org.testng.internal.thread.graph.IWorker<ISuite> ```

An `IWorker` that is used to encapsulate and run Suite Runners

**Author:**
:   cbeust, nullin

---

**Constructor Summary**

`SuiteRunnerWorker(ISuite suiteRunner,
org.testng.internal.SuiteRunnerMap suiteRunnerMap,
int verbose,
java.lang.String defaultSuiteName)`


| **Method Summary** | |
| --- | --- |
| `int` | `compareTo(org.testng.internal.thread.graph.IWorker<ISuite> arg0)` |
| `int` | `getPriority()` |
| `java.util.List<ISuite>` | `getTasks()` |
| `long` | `getTimeOut()` |
| `void` | `run()` |
| `java.lang.String` | `toString()` |

**Methods inherited from class java.lang.Object**

`clone, equals, finalize, getClass, hashCode, notify, notifyAll, wait, wait, wait`

**Constructor Detail**

### SuiteRunnerWorker

```
public SuiteRunnerWorker(ISuite suiteRunner,
                         org.testng.internal.SuiteRunnerMap suiteRunnerMap,
                         int verbose,
                         java.lang.String defaultSuiteName)
```


**Method Detail**

### run

```
public void run()
```

:   **Specified by:**: `run` in interface `java.lang.Runnable`

---


### compareTo

```
public int compareTo(org.testng.internal.thread.graph.IWorker<ISuite> arg0)
```

:   **Specified by:**: `compareTo` in interface `java.lang.Comparable<org.testng.internal.thread.graph.IWorker<ISuite>>`

---


### getTasks

```
public java.util.List<ISuite> getTasks()
```

:   **Specified by:**: `getTasks` in interface `org.testng.internal.thread.graph.IWorker<ISuite>`

---


### toString

```
public java.lang.String toString()
```

:   **Overrides:**: `toString` in class `java.lang.Object`

---


### getTimeOut

```
public long getTimeOut()
```

:   **Specified by:**: `getTimeOut` in interface `org.testng.internal.thread.graph.IWorker<ISuite>`

---


### getPriority

```
public int getPriority()
```

:   **Specified by:**: `getPriority` in interface `org.testng.internal.thread.graph.IWorker<ISuite>`


---


|  |  |  |
| --- | --- | --- |
| **Overview** \| **Package** \| **Class** \| **Tree** \| **Deprecated** \| **Index** \| **Help** | |  |
| **PREV CLASS**   **NEXT CLASS** | **FRAMES**    **NO FRAMES**     **All Classes** |  |
| SUMMARY: NESTED \| FIELD \| CONSTR \| METHOD | DETAIL: FIELD \| CONSTR \| METHOD |  |


---
